# Self-assembly of nucleic acid molecular aggregates catalyzed by a triple-helix probe for miRNA detection and single cell imaging†

**DOI:** 10.1039/c6sc00694a

**Published:** 2016-03-07

**Authors:** Zhen Zhang, Yuanyuan Wang, Ningbo Zhang, Shusheng Zhang

**Affiliations:** a Shandong Province Key Laboratory of Detection Technology for Tumor Makers, College of Chemistry and Chemical Engineering, Linyi University Linyi 276000 P. R. China shushzhang@126.com; b Collaborative Innovation Center of Functionalized Probes for Chemical Imaging in Universities of Shandong, Shandong Normal University Jinan 250014 P. R. China

## Abstract

We herein report a novel finding that nucleic acid molecular aggregates (NAMAs) self-assembled on graphene oxide nanoplates (GONPs) as a result of DNA rolling circle amplification (RCA) and a functionalized triple-helix probe (THP) in single cells. The functionalized THP containing the aptamer region for target recognition and the trigger DNA region for RCA was firstly used to activate RCA for miRNA imaging in single cells. Interestingly, NAMAs with the fluorescent labels were hybridized by both the RCA products and FAM-DNA, and could partly self-assemble on GONPs; meanwhile, NAMAs could extend from the GONPs, which led to the quenched fluorescence being renewed. Significantly, the NAMAs were successfully applied for low-abundance miRNA detection and imaging in single cells. The self-assembled NAMAs could generate prominent and agminated fluorescence-bright spots in single cancer cells, which will effectively drive cell imaging into a new era.

## Abstract 2

We developed a new and highly sensitive method for miRNA detection and intracellular imaging based on novel nucleic acid molecular aggregates self-assembled on graphene oxide nanoplates.

## Graphical abstract

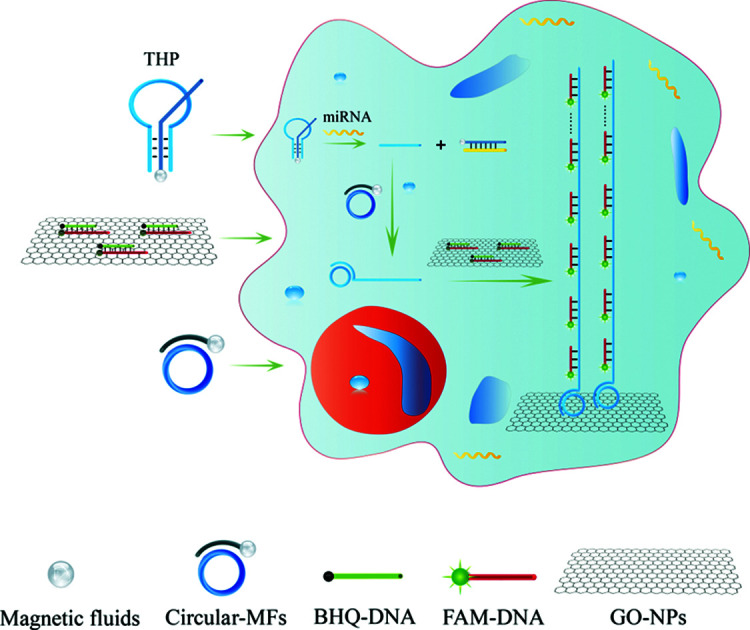

## Introduction

Cancer is a pervasive and devastating illness that influences the lives of millions of people annually.^1–4^ If cancer is diagnosed at an early stage, the chance of survival for patients can be tremendously enhanced. Recognizing cancer at the cellular level before cancerization occurs holds great promise for enhancing the survival rates of carcinoma patients. The major challenge of this study is the assessment of abnormalities in gene expression in intact cancer cells.^5–9^ Cancer-related miRNAs have been regarded as special biomarkers for estimating the migration of tumor cells. Alterations in cancer-related miRNA expression levels are associated with tumor burden and malignant progression.^10–13^ The detection of cancer-related miRNAs in single tumor cells offers an appealing tool for recognizing cancer cells in clinical samples. Nevertheless, it is difficult to analyze cancer-related miRNAs in living cells due to their unique characteristics, such as their small size, low abundance in total RNA samples, and sequence homology among family members.^14–17^

Recent advances in nanotechnology have helped in the development of intracellular analysis, single cell monitoring and imaging.^18,19^ Notably, nanoparticles functionalized with aptamers play an essential role in a specific subset of cells. It was reported that 2 h of incubation was quite sufficient for the internalization of nanoparticles into live cells.^20,21^ Moreover, DNA-conjugated and carboxyl-modified magnetic fluids (CMFs) were quickly taken up by the cells owing to their targeted binding and endocytosis. This ultimately led to a higher internalization level of CMFs into cells within 2 h, compared to the non-targeted nanoparticles.^22^ Meanwhile, CMFs could prevent side reactions in the process of preparing the probe through magnetic separation. Considering the validity of CMFs, we chose probe-conjugated CMFs for future experiments. In addition, graphene oxide (GO) has displayed excellent application potential, for example GO may act as a transporter of genes into cells and a sensing platform with excellent fluorescence quenching, and may provide effective protection of oligonucleotides from enzymatic cleavage while transmitting them to intracellular spaces.^23–25^ Furthermore, GO can interact strongly with single-stranded DNA (ssDNA), whereas it has less affinity toward double-stranded DNA (dsDNA).^26^ Nonetheless, the exploration of GO for living cell analysis still remains at an early stage. The challenges in the application of GO were mainly the restricted sensitivity for intracellular analysis, live cell imaging, and detection with a nanomolar level detection limit.^24,27,28^ These shortcomings are primarily attributed to the lack of signal amplification and mechanisms for gathering signal-molecules in these methods. Thus, strategies for the specific, sensitive and quantitative detection of miRNAs in living cells are of vital significance.

Aiming to explore the unrevealed sensing potential for early cancer detection and conquer the existing challenges, we firstly developed a new and highly sensitive method for miRNA detection and intracellular imaging based on novel nucleic acid molecular aggregates (NAMAs) self-assembled on graphene oxide nanoplates (GONPs). A functionalized THP containing the aptamer region for target recognition and the trigger DNA region for RCA was introduced into single cells to activate the RCA reaction for the first time. Interestingly, NAMAs labeled with 6-carboxylfluorescein (FAM) were hybridized by both the RCA products and FAM-DNA, and were a complex of dsDNA and ssDNA. The NAMAs could partly self-assemble on the GONPs, and in the meantime NAMA-FAM could extend from the GONPs, which led to the quenched FAM being renewed. Significantly, NAMAs were successfully applied for low-abundance miRNA detection and imaging in single cells. In contrast to conventional RCA, the self-assembled NAMAs could generate agminated and prominent fluorescence-bright spots in single cancer cells, which may significantly distinguish between cancer cells and normal cells. Such an original intracellular imaging technique for miRNAs based on NAMAs may promote the progress of early cancer detection, which will spur on the development of a new and effective technology for visual cell research.

## Results and discussion

### Design for miRNA detection and imaging

**Scheme 1:**
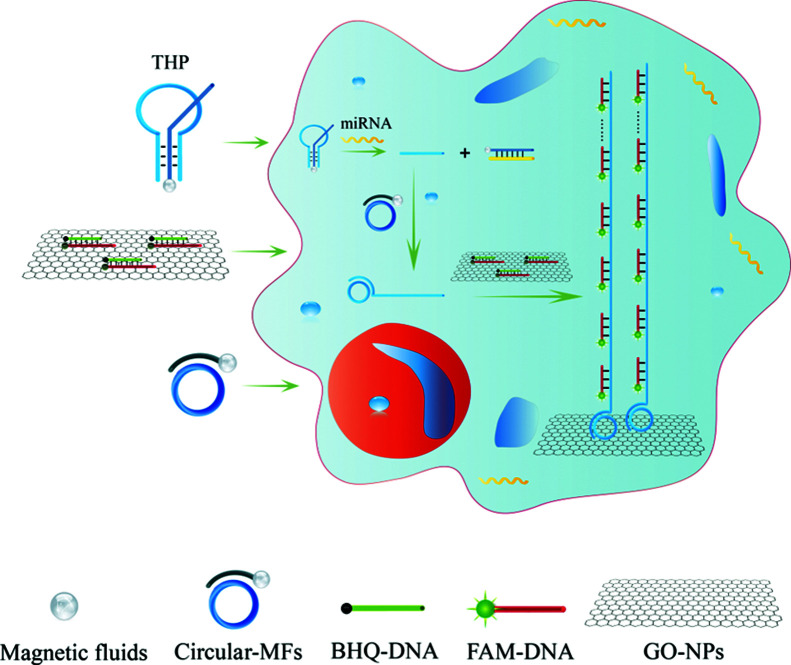
Schematic representation of miRNA detection and visualizing based on NAMAs in single cells.

**Scheme 2:**
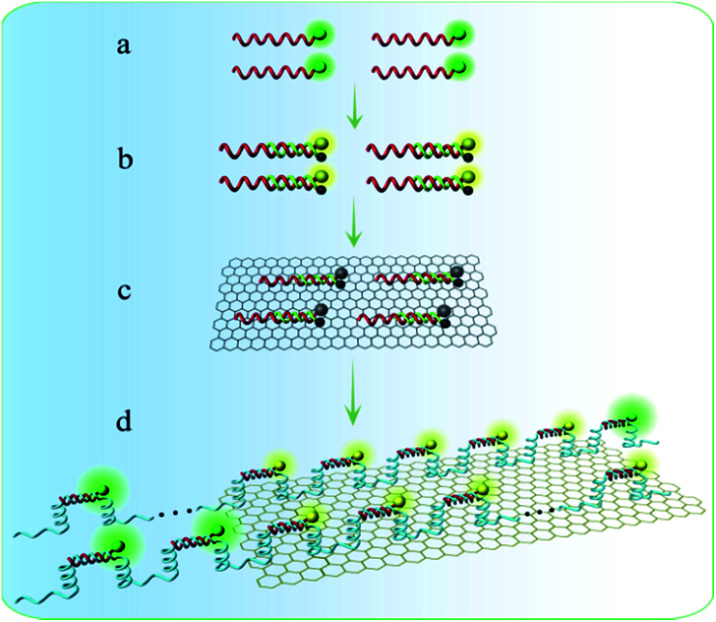
Schematic illustration of fluorescence in the four different states. (a) FAM-DNA, (b) the product DHS hybridized with FAM-DNA and BHQ-DNA, (c) DHS on GONPs, (d) NAMA-FAM (the complex hybridized with the RCA products and FAM-DNA) self-assembled on GONPs.

In this work, we constructed novel NAMA-FAM for analyzing target miRNA in single cells based on a functionalized THP probe and RCA (Scheme 1). Firstly, the THP probe conjugated onto CMFs was ingeniously designed and could serve not only as a probe to specifically identify cancer-related miR-21 but also as a primer for the RCA reaction in single cells. Secondly, after the THP-CMF probe enters into cells, miR-21 can combine with the THP-CMF probe. Meanwhile, the primer DNA1 strands were substituted and released from the THP probe, to activate the intracellular RCA reaction in the presence of circular DNA3. Thirdly, GONPs loaded with many fully quenched DHS strands (dsDNA hybridized segmentally by FAM-DNA5 and BHQ-DNA6, abbreviated as DHS) were incubated into single cells. Herein, BHQ and GONPs were simultaneously employed to avoid interference in subsequent experiments (Fig. S2†). Finally, FAM-DNA from the quenched DHS hybridized with the RCA products. NAMA-FAM self-assembled on GONPs was generated (Scheme 2d), and single cells displayed prominent and agminated fluorescence-bright spots. Thus, miRNA detection and imaging in the single cells was successfully achieved.

**Fig. 1:**
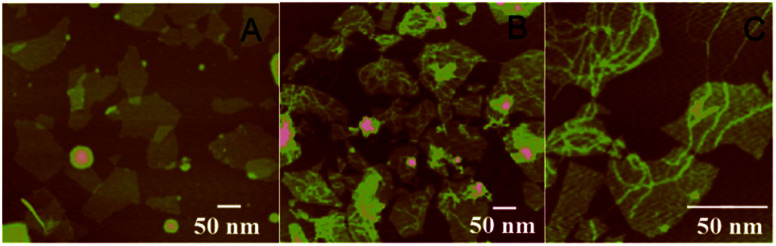
(A) AFM images of GONPs, (B and C) AFM images of NAMAs self-assembled on GONPs.

A key design in the intracellular strategy was the novel nano-assembly of NAMA-FAM on GONPs (Scheme 2). BHQ-DNA can partly quench FAM-DNA (Scheme 2b). The adsorption selectivity of different nucleic acids on the GONP surface was studied through fluorescence detection. The results are shown in Fig. S1.† The GONPs could adsorb the ssDNA sections of DHS, resulting in FAM-DHS being adequately quenched (Scheme 2c). Interestingly, NAMA-FAM could partly self-assemble on the GONPs, while simultaneously, NAMA-FAM extended from the GONPs. Atomic force microscopy (AFM) was used to confirm the envisaged morphology of the self-assembled NAMA-GONP structure, compared with the AFM images of the GONPs, as shown in Fig. 1. Dramatically, the quenched FAM could be renewed (Scheme 2d). FAM-DNA was separated from BHQ-DNA (yellow-green) and hybridized with the RCA products (baby blue), which led to the quenching by BHQ disappearing and the distance altering between FAM and the GONPs. In addition, a part of the NAMAs self-assembled on the GONPs might not be fully quenched, due to the GONPs interacting strongly with the ssDNA of the NAMAs and having less affinity toward the dsDNA sections of the NAMAs.^26^ The length of the NAMAs might be longer than the GONPs (Scheme 2d), so that the parts of the NAMAs that might extend from the GONPs could not be quenched (Fig. 1C).

### Characterization of the formation of the THP molecules

**Fig. 2:**
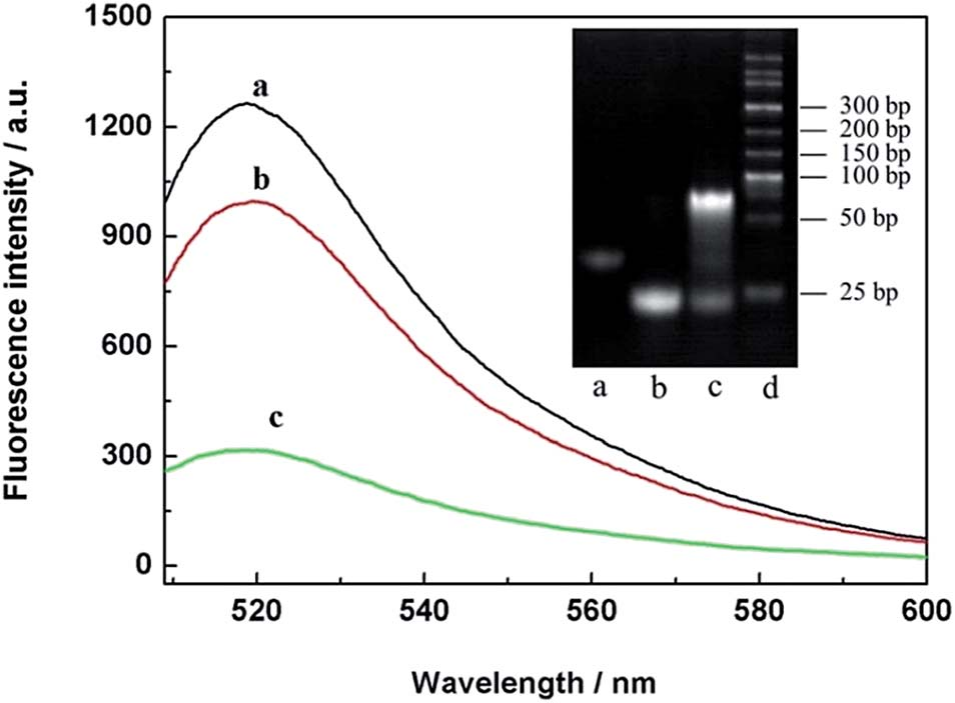
Fluorescence responses of FAM-DNA7 (a), noncomplementary BHQ-DNA9 and FAM-DNA7 (b) and complementary BHQ-DNA8 and FAM-DNA7 (c). Inset: the THP molecules were analyzed using polyacrylamide gel electrophoresis, (a) DNA1, (b) DNA2, (c) THP molecules, and (d) the marker.

To verify the THP molecules, a series of contrast experiments were carried out. The results are shown in Fig. 2. When FAM-DNA7 (curve a) was presented alone, there was a relatively high fluorescence intensity. The fluorescence intensity showed little change when FAM-DNA7 and non-complementary BHQ-DNA9 were presented at the same time (curve b). But in the presence of FAM-DNA7 and complementary BHQ-DNA8, the fluorescence intensity sharply decreased (curve c). The main reason was that the distance between the fluorophore and quencher was diminished through the hybridization of T–A·T and C–G·C base triplets.^29^ Meanwhile, the THP molecules were analyzed *via* polyacrylamide gel electrophoresis (15%, 110 V, 70 min), stained with ethidium bromide (EB), as shown in the inset of Fig. 2. According to the results of the fluorescence characterization and polyacrylamide gel electrophoresis, the THP molecules were fabricated successfully.

### The cytotoxicity tests of the THP-CMF probe and GONPs

**Fig. 3:**
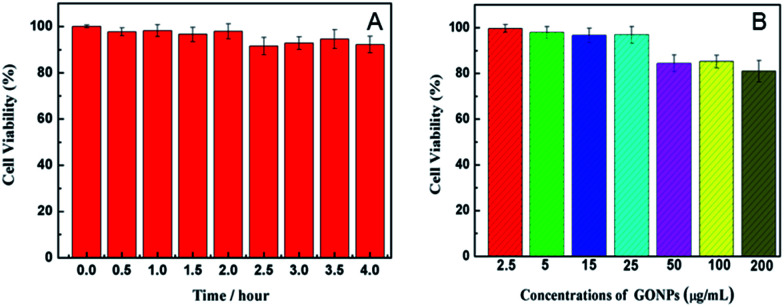
(A) Viability of HeLa cells (100 μL, 1.0 × 10^6^ mL^−1^) after incubation with 30 μL of the THP-CMF probe for different time periods. (B) Viability of HeLa cells after incubation with different concentrations of GONPs.

The cytotoxicity tests of the THP-CMF probe and GONPs were respectively studied with HeLa cells by MTT experiment,^30,31^ as shown in Fig. 3. After the cells were incubated with 200 μL of culture medium containing 30 μL of the THP-CMF probe for different time periods and were washed once with 200 μL of PBS (0.01 M, pH 7.4), MTT (100 μL, 0.5 mg mL^−1^) was seeded in the wells and incubated at 37 °C for 4 h. Afterwards, 150 μL of DMSO was added to each well to dissolve the crystals composed of living cells, and the absorbance at 490 nm was tested to determine the relative cell viability. The test showed that HeLa cells maintained about 91.6% of the cell viability by (*A*_test_/*A*_control_) × 100% after incubation with 30 μL of the probe for 4 h, demonstrating that the designed THP-CMF probe possessed low cytotoxicity.

After the cells were incubated with 200 μL of culture medium containing different concentrations of GONPs for 14 h, then were washed two times with 200 μL of PBS, 100 μL of MTT solution was added to each well. After 4 h, 150 μL of DMSO was added to each well and the absorbance at 490 nm was tested to determine the relative cell viability by (*A*_test_/*A*_control_) × 100%, as shown in Fig. 3B. The experiment displayed that HeLa cells could maintain about 96.2% of the cell viability after incubation with 25 μg mL^−1^ GONPs for 14 h, indicating a satisfactorily low cytotoxicity.

### Immobilization of THP molecules and circular-DNA onto CMFs

The immobilization of THP molecules on the surface of CMFs (Fig. S3†) was achieved with some modifications of the literature procedure for the fastening of DNA chains onto carboxyl-modified magnetic beads.^21,22^ Firstly, 70 μL of CMFs was washed with 200 μL of 0.1 M imidazole-HCl solution (pH 6.8) three times. The CMF suspensions were separated from the solution using a magnetic rack. Secondly, to activate the carboxyl groups on the CMFs, the CMFs were incubated at 37 °C for 30 min in imidazole-HCl solution (pH 7.4, 0.1 M, 200 μL) containing 0.1 M EDC. Then the CMFs were rinsed with 200 μL of PBS (0.01 M, pH 7.4) three times. Thirdly, 200 μL of 1.0 × 10^−6^ M DNA2 modified with amino groups was added to the freshly activated CMFs and incubated at 37 °C for 12 h. Fourthly, the excess DNA was removed using magnetic separation. DNA2 strands linking on the surface of the CMFs (DNA2-CMF) were washed three times with 200 μL of PBS, then dispersed in 200 μL of PBS. Fifthly, DNA1 strands (400 μL, 1.0 × 10^−6^ M) were added into the dispersed liquid of DNA2-CMF, and then were incubated at 37 °C for 3 h. Finally, after the excess DNA strands were removed using magnetic separation, the functional THP-CMF probe was successfully obtained (Fig. S4†). Similarly, the immobilization of DNA4 on the surface of CMFs was achieved following the procedure described above, then circular DNA3 and DNA4-CMF were incubated at 37 °C for 4 h. CMF-circular-DNA was well fabricated.

### Detection capability for miRNA

**Fig. 4:**
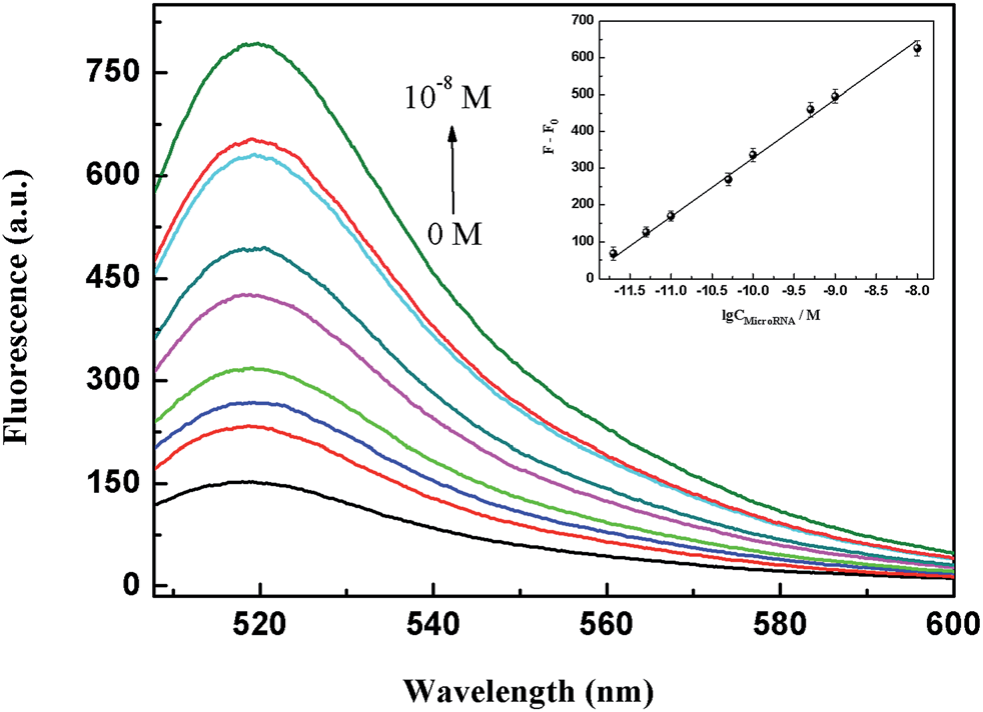
Fluorescence spectral responses to target miRNA of varying concentrations *in vitro*. miR-21 concentrations: 0 M, 2.0 × 10^−12^ M, 5.0 × 10^−12^ M, 1.0 × 10^−11^ M, 5.0 × 10^−11^ M, 1.0 × 10^−10^ M, 5.0 × 10^−10^ M, 1.0 × 10^−9^ M and 1.0 × 10^−8^ M. Inset: the corresponding calibration curve of the fluorescence intensity *versus* the concentration of miR-21. The average of three spectra was acquired for different detection, and three repetitive experiments were carried out. The error bars display the standard deviation of the three experiments. The blank was deducted from each value.

In the following experiment *in vitro*, we utilized a unique function of GONPs to protect the target miR-21 from enzymatic digestion effectively, attributing the fact that single-stranded RNA (ssRNA) could bind strongly to the GONP surface through π–π stacking to a steric hindrance effect.^32^ Under the optimum conditions (for details of the optimization experiments see the ESI†), the fluorescence intensity increased with the addition of the different miR-21 concentrations shown in Fig. 4. The regression equation was described as: *F* − *F*_0_ = 156.6 lg *C* + 1895.5 (*F* represents the fluorescence intensities of the amplification products in the presence of miR-21; *F*_0_ represents the fluorescence intensities in the absence of miR-21; *C* represents the concentration of miR-21), and the corresponding correlation coefficient (*R*) of the calibration curve was 0.997. The reproducibility of the THP-NAMA system was examined using 11 successive measurements of 5.0 × 10^−10^ M miR-21 under the optimum conditions. The relative standard deviation (RSD) was calculated to be 9.7%, suggesting the good reproducibility of this method.

### Cell imaging of miRNA with NAMAs self-assembled on GONPs

After verification *in vitro*, we explored the potential of NAMAs self-assembled on GONPs for imaging miR-21 in single cells. For the imaging experiments, four types of cells (HepG2, MCF-7, HeLa and L-02) were separately cultured in 6-well slides. Firstly, the slides with fixed cells were incubated with culture medium containing DHS on GONPs (25 μg mL^−1^) for 12 h, then were washed three times with PBS (0.01 M, pH 7.4). Secondly, the THP probe and circular-DNA fastened onto CMFs were incubated with the cells. The DNA-conjugated CMFs were quickly taken up by the cells owing to the targeted binding and endocytosis which ultimately led to a higher level of CMF internalization into the cells within 2 h.^20–22^ The slides with fixed cells were incubated with 2% formamide for 30 min. After being washed three times with PBS, the slides with fixed cells were dehydrated using a series of 70%, 80%, and 99% ice-cold ethanol for 3 min each and air-dried. Thirdly, the RCA reaction was performed in a humidified 37 °C incubator for 90 min with the reaction solution containing DEPC-treated water, 2 μL of Phi29 DNA polymerase and 6 μL of dNTPs.^33–35^ Then, all the imaging processes were performed at 37 °C within 4 h. The fluorescence-bright spots could be visualized using confocal microscopy.

**Fig. 5:**
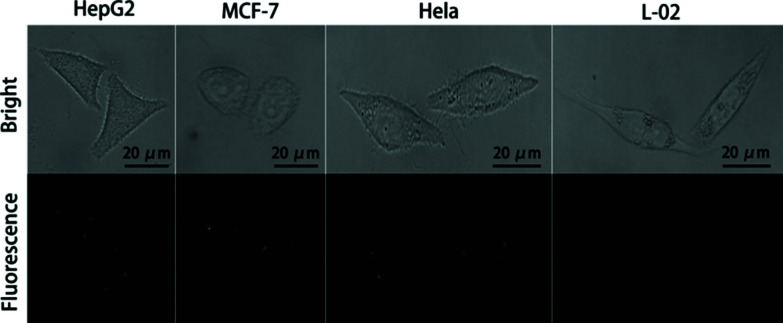
Evaluation of NAMAs self-assembled on GONPs for target miR-21 imaging in single cells (HepG2, MCF-7 cells, Hela and L-02 cells).

Single miR-21 molecules were extended to long chains including a mass of tandem repeats by RCA, then the long chains hybridized with fluorescent probes (FAM-DNA5). Interestingly, in contrast with conventional RCA, due to the synergy between RCA and the NAMAs self-assembled on GONPs, they could self-assemble into aggregated, unique and larger fluorescence-bright spots in single cells, which demonstrated that low-abundance miRNA imaging in single tumor cells was feasible using the THP-NAMA method. In Fig. 5, the larger fluorescence-bright spots can be vividly observed in single MCF-7, HepG2 and HeLa cells. However, these great fluorescence-bright spots were not seen in human normal hepatocytes (L-02), and small and scattered fluorescence spots were very rare. Because the expression level of miR-21 in normal L-02 cells was very low, NAMAs labeled with FAM could hardly take shape. These results indicated that the relative expression levels of tumor-related miRNA in single MCF-7, HepG2 and HeLa cells were higher than in L-02 cells. Thus, the new method was viable for detecting changes in gene expression levels in single tumor cells.

## Experimental section

### Reagents and materials

MCF-7 and HeLa cells were purchased from the KeyGEN biotechnology Company (Nanjing, China). The human hepatocellular liver carcinoma cell line HepG2 was purchased from the Shanghai Bioleaf Biotechnology Company (Shanghai, China), and human normal hepatocytes L-02 were from Silver Amethyst Biotech. Co. Ltd (Beijing, China). A mirVana miRNA isolation kit and fetal bovine serum were purchased from Life Technologies (Carlsbad, California). Dimethyl sulphoxide (DMSO), 3-(4,5-dimethyl-thiazol-2-yl)-2,5-diphenyltetrazolium bromide (MTT), carboxyl PEG (MW 250), and oleic acid were purchased from Sigma Chemical Company; K_2_S_2_O_8_, P_2_O_5_, KMnO_4_ and graphite flakes were ordered from Aladdin Industrial, Inc. (Shanghai, China); Phi29 DNA polymerase, diethylprocarbonated (DEPC)-treated deionized water and a deoxynucleotide solution mixture (dNTPs) were ordered from TaKaRa Biotechnology Co., Ltd (Dalian, China). Gel electrophoresis loading buffer and ladder DNA were purchased from Solarbio. Co. Ltd (Beijing, China). Phosphate buffer saline (PBS, pH 7.4) contained 136.7 mM NaCl, 2.7 mM KCl, 8.72 mM Na_2_HPO_4_, and 1.41 mM KH_2_PO_4_. All reagents in this work were of analytical grade, which were used as received unless otherwise mentioned. DEPC-treated deionized water was used in all experiments. DNA oligonucleotides (except circular-DNA) were purchased from Sangon Biotech Co., Ltd (Shanghai China). Circular-DNA and MicroRNA sequences were synthesized and purified by TaKaRa Biotechnology Co., Ltd (Dalian, China). Their detailed sequences are listed in Tables S1 and S2.†

### Instruments

Fluorescence imaging was performed using a Leica TCS SP8 inverted confocal microscope (Leica, Germany). The cellular images were acquired using a 100× objective. A solid laser (488 nm) was used as the excitation source for the FAM-labeled probe, and a 495–545 nm bandpass filter was used for fluorescence detection. AFM images were taken on a NanoScope IIIa MultiMode AFM (Veeco, USA). All fluorescence measurements were carried out on a F4600 fluorometer (Hitachi, Japan). The emission spectra were obtained by exciting the samples at 492 nm and scanning the emission from 450 to 600 nm.

### Synthesis and characterization of GONPs

GONPs were prepared using the Hummers and Hoffman method with some modifications,^36–38^ followed by strong sonication and centrifugal separation to disperse and remove large GO layers. Firstly, graphite flakes (2.0 g) crushed using an ultrasonic cell disruptor were mixed with 15 mL of concentrated H_2_SO_4_, 3.0 g of K_2_S_2_O_8_ and 3.0 g of P_2_O_5_, and were then incubated at 80 °C using an oil-bath and kept stirring for 6 h. The pretreated graphite was naturally cooled to ambient temperature and diluted with ultrapure water. Secondly, the mixture was acquired by filtering using a 0.2 micron Nylon film until neutral and dried. The pre-oxidized graphite was then re-oxidized using the Hummers' method. The product was placed in concentrated H_2_SO_4_ at 0 °C. Subsequently, 20 g of KMnO_4_ was added gradually with stirring, meanwhile, the temperature of the mixture was kept around 10 °C using an ice-bath. The mixture was stirred at 35 °C for 4 h, followed by the injection of ultrapure water, and the stirring was continued for 30 min. Thirdly, 800 mL of ultrapure water was instilled into the mixture followed by the addition of 30% H_2_O_2_ (30 mL) drop by drop. The mixture was filtered and washed with 10% HCl solution to remove the metal species. Afterwards, the sediment was washed with ultrapure water and centrifuged repeatedly until the solution became neutral. The sample was dried in air and diluted to give a 1% (w/w) graphite oxide dispersion. Finally, this dispersion was purified using dialysis for about 7 days to remove residual metal ions. To exfoliate the oxidized graphite, the product was sonicated 3 times, 30 min/time, and then centrifuged at 12k rpm for 15 min. The exfoliated GONPs were acquired from the supernatant.

### Cell culture and miRNA preparation

MCF-7, HepG2, HeLa and L-02 cells were separately cultured in RPMI 1640 (Hyclone, penicillin 100 U mL^−1^ and streptomycin 100 μg mL^−1^) plus 10% fetal bovine serum and maintained at 37 °C in a humidified atmosphere containing 5% CO_2_, according to the instructions of the American Type Culture Collection. MCF-7 cells were chosen as a representative for measuring the intracellular miR-21 level. MCF-7 cells were collected and centrifuged at 3000 rpm for 6 min in a culture medium, rinsed once with PBS buffer, and then spun down at 3000 rpm for 6 min. The cell pellets were suspended in 700 μL of lysis solution. Total RNA was extracted from the MCF-7 cells using the mirVana miRNA isolation kit according to the manufacturer's procedure. The sample of miR-21 from these cells was diluted and then analyzed for the subsequent miRNA experiments.

### *In vitro* experiments for target miR-21, and the preparation of NAMAs self-assembled on GONPs

For measuring the intracellular miR-21 level, total RNA was extracted from MCF-7 cells using the mirVana miRNA isolation kit according to the manufacturer's procedure. Firstly, 100 μL of the functional THP-CMF probe and target miR-21 of varying concentrations were incubated at 37 °C for 3 h, then the clear liquid Q1 was obtained using magnetic separation. Secondly, 100 μL of circular-DNA-CMF, the clear liquid Q1, dNTPs and 0.2 U μL^−1^ of Phi29 DNA polymerase were incubated at 37 °C for 3 h, and then the clear liquid Q2 was acquired through magnetic separation. Thirdly, 25 μg mL^−1^ GONPs loaded with many fully quenched DHS strands (DHS hybridized with BHQ-DNA and FAM-DNA) and the clear liquid Q2 were incubated at 37 °C for 3 h, then centrifuged and washed three times to obtain NAMAs self-assembled on GONPs.

## Conclusions

In summary, we have successfully demonstrated the concept of NAMAs self-assembling on GONPs as a result of DNA rolling circle signal amplification. RCA was first activated by the difunctional triple-helix probe in single cells for target miRNA detection. Importantly, the NAMAs self-assembled on GONPs were successfully applied for low-abundance miRNA imaging in single cells, which could form unique, large and congregated fluorescence-bright spots for significantly distinguishing between cancer cells and normal cells. Thus, our findings on NAMAs will likely open the door to gaining new insights in the field of imaging, aiding clinical diagnosis and the detection of specific nucleic acid sequences.

## Supplementary Material

SC-007-C6SC00694A-s001
