# Supplementary material for: Self-assembly of nucleic acid molecular aggregates catalyzed by a triple-helix probe for miRNA detection and single cell imaging
Source: Chem Sci. 2016 Mar 7;7(7):4184–9. doi: 10.1039/c6sc00694a (PMC6013927; doi:10.1039/c6sc00694a)
Supplement: SC-007-C6SC00694A-s001 [file SC-007-C6SC00694A-s001.pdf]

Electronic Supplementary Material (ESI) for Chemical Science.
This journal is © The Royal Society of Chemistry 2016

Supplementary Information

Self-assembled Nucleic Acids Molecular Aggregates Catalyzed by a Triple-helix Probe for miRNA Detection and Visualizing in Single Cells

Zhen Zhang,[a] Yuanyuan Wang,[b] Ningbo Zhang,[a] Shusheng Zhang[a] *

[a] Shandong Province Key Laboratory of Detection Technology for Tumor Makers, College of Chemistry and Chemical Engineering, Linyi University, Linyi 276000, P. R. China.

[b] Collaborative Innovation Center of Functionalized Probes for Chemical Imaging in Universities of Shandong, Shandong Normal University, Jinan 250014, P. R. China.

*Corresponding author. Fax: +86-539-8766107; Tel: +86-539-8766107

E-mail: shushzhang@126.com;

S1. Adsorption Selectivity of GONPs for Different Types of Nucleic Acids.

A series of control experiments were put into effect in PBS (0.01M, pH 7.4). The results were exhibited in Fig. S1. In the presence of FAM-DNA5 (curve a), the fluorescence intensity was relatively high. In the presence of FAM-DNA5 and BHQ-DNA6, the fluorescence intensity was reduced about 3.4 times (curve b), since BHQ-DNA6 could hybridize with FAM-DNA5 to partially quench the fluorescence signal. In the presence of GONPs, the fluorescence intensity was dramatically reduced (curve c), because GONPs can interact strongly with ssDNA section of the hybridized product and quench fully the fluorescence signal, leading to the fluorescence intensity decreasing about 12.2 times, compared with the fluorescence intensity of FAM-DNA5.

BHQ-DNA and GONPs were simultaneously employed to avoid the interference in rolling circle amplification experiments. GONPs can interact strongly with ssDNA-FAM and then quench the fluorescence in PBS (0.01M, pH 7.4), nevertheless,

a little of interference might appear in the reaction solution for rolling circle amplification (RSRCA), which would lead to blank value being mildly increased (Fig. S2-c). It might be a reason that a very little of ssDNA-FAM scattered from GONPs. So, FAM-DNA5 and BHQ-DNA6 hybrids (DHS) were used for reducing blank value, because fluorescence intensity by a very little of DHS scattered from GONPs was very weak (Fig. S2-e). Meanwhile, only when the RCA reaction occurred in the system, BHQ-DNA6 could be replaced by RCA-product, FAM-DNA5 and RCA-product hybrids could generate and fluoresce, which could avoid the interference.

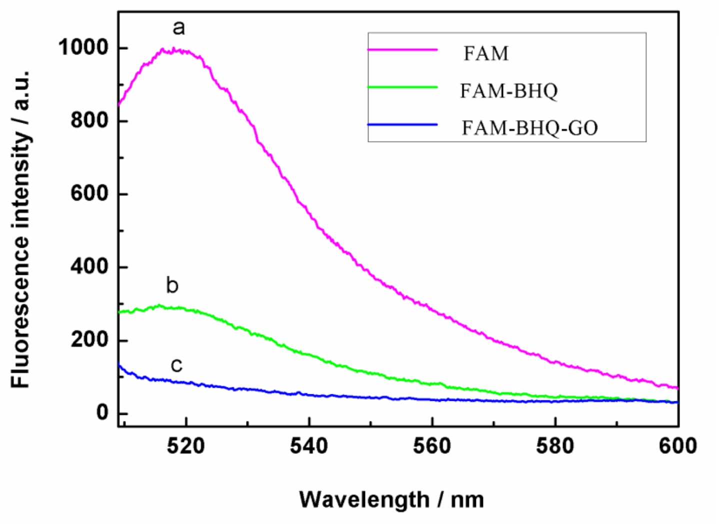

Fig. S1 Fluorescence responses of FAM-DNA5 (a), the product DHS hybridized by FAM-DNA5 and BHQ-DNA6 (b), DHS on GONPs (c).

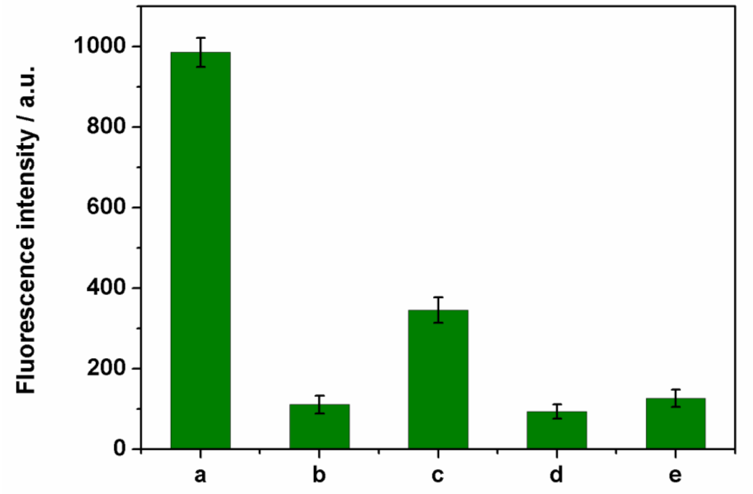

Fig. S2 Fluorescence responses of FAM-DNA5 in PBS (a), FAM-DNA5 on GONPs in PBS (b) and in RSRCA (c), DHS on GONPs in PBS (d) and in RSRCA (e).

S2. Carboxyl-modified Magnetic Fluids (CMFs).

CMFs were prepared through co-precipitation of Fe (II) and Fe (III) [S1-S3]. In brief, $FeCl_2{\bullet}4H_2O$(0.01 mol) and $FeCl_3{\bullet}6H_2O$ (0.02 mol) were dissolved in 80 ml of water in a round bottom flask and temperature was slowly increased to 70 ℃ in nitrogen atmosphere under refluxing condition with a constant mechanical stirring of 1000 rpm. The temperature was maintained at 70 ℃ for 30 min and then 30 ml of 25% ammonia solution was added instantaneously to the above reaction mixture and stirred for another 30 min at the same temperature. Then, oleic acid (5.65 g) was added dropwise and reacted at 70 ℃ for 90 min under stirring. The precipitates were thoroughly washed distilled water and separated from the supernatant solution by magnetic decantation. The obtained CMFs (Fig. S3) were dispersed uniformly by ultrasonics and stored at 4 ℃.

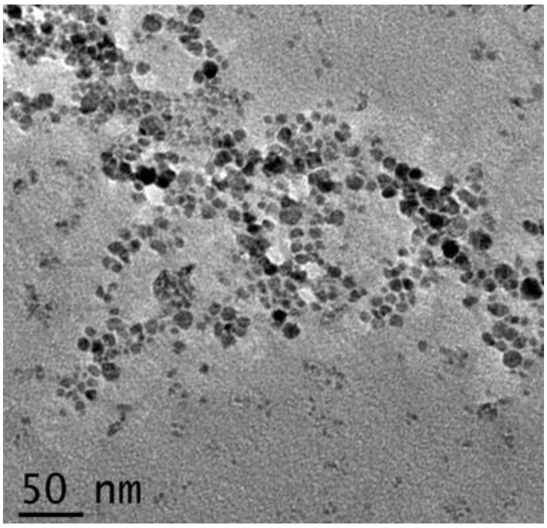

Fig. S3 TEM images of CMFs (10 nm).

S3. The Triple-helix Probes Immobilized on Carboxyl-modified Magnetic Fluids

The THP molecules immobilized on the surface of CMFs were achieved by some modifications according to the literature that DNA chains were fastened on carboxyl-modified magnetic beads.[S4, S5] 80 μL 1.0 $\times 10^{-6}$ M DNA2 modified amino

group were added to the activated CMFs and incubated at 37 ℃ for 12 h. The excess DNA was moved away by magnetic separation. DNA2 strands linking on the surface of CMFs (DNA2-CMFs) were washed three times by PBS, then dispersed in 200 μL PBS. Fifth, FAM-DNA10 (80 μL, 1.0×10^{-6} M) were added into the dispersed liquid of DNA2-CMFs, then were incubated at 37 ℃ for 3h. Finally, the functional THP-CMFs probe was successfully obtained by magnetic separation, and liquid supernatant after magnetic separation was gained. Compared with fluorescence intensity of FAM-DNA10 (curve a), fluorescence intensity of liquid supernatant after magnetic separation of the THP-CMFs probes (curve b) reduced obviously (Fig. S4). It showed that FAM-DNA10 could well hybridize with DNA2-CMFs to form THP-CMFs probe. According to the result of fluorescent characterization and polyacrylamide gel electrophoresis (Fig.2), the THP molecules could be fabricated successfully. These results indicated the triple-helix probes could be well immobilized on carboxyl-modified magnetic fluids.

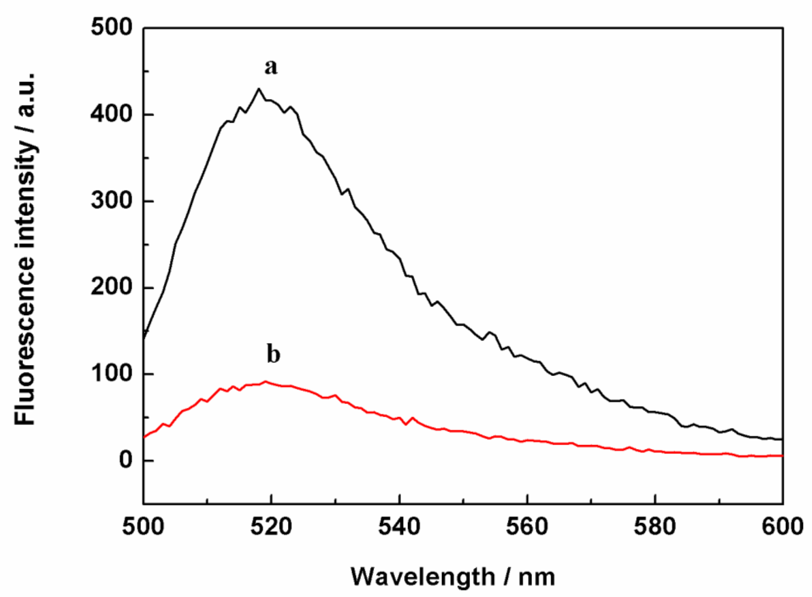

Fig. S4 Fluorescence spectral responses. (a) FAM-DNA10, (b) Liquid Supernatant after magnetic separation of the THP-CMFs probes.

S4. Optimization of the experimental conditions and gel electrophoresis analysis of the RCA products

The amount of Phi29 DNA polymerase was an important influencing factor for the

fluorescence intensity. To improve the sensitivity of fluorescence detection, a series of control experiments were designed to optimize the amount of Phi29 DNA polymerase. As shown in Fig. S5-A, the fluorescence intensities enhanced speedily when the amount of polymerase increased from 0.05 to 0.2 UμL^{-1}. But after 0.2 UμL^{-1}, the fluorescence intensity changed mildly. Thus, 0.2 UμL^{-1} of Phi29 DNA polymerase was chosen to be the optimum. For obtaining the best sensing performance, the incubation time was investigated by implementing the tests of different reaction time. As illustrated in Fig. S5-B, the fluorescence intensity boosted rapidly with the increase of incubation time, and gained a plateau after 180 min. So the incubation time of ring rolling reaction was considered to be at 180 min. The RCA reaction was analyzed by 1.0% agarose gel electrophoresis, stained with EB, as shown in Fig. S5-C.

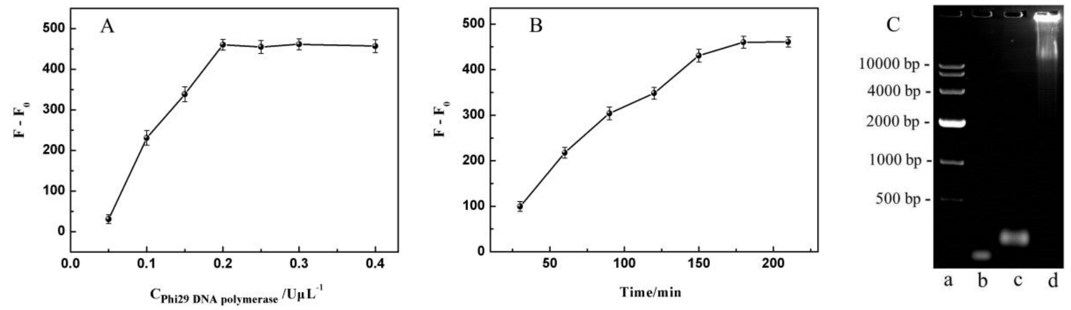

Fig. S5 (A) Influence of the amount of Phi29 DNA polymerase on the fluorescence intensity respond of 5.0 $\times 10^{-10}$ M miR-21. (B) Effect of the incubation time on the fluorescence intensity respond of 5.0 $\times 10^{-10}$ M miR-21. (C) The RCA reaction was analyzed by 1.0% agarose gel electrophoresis, (a) the marker, (b) DNA3, (c) hybridization of DNA4 and DNA3, (d) the RCA product.

S5. Specificity of the assay

It is a great challenge to put into effect the miRNAs assay with high specificity owing to the high sequence homology among family members and small size of miRNAs. So the selectivity of the THP-NAMAs strategy was investigated by four different miRNAs (miR-21, let-7a, let-7b, and let-7c) with the same concentration. Fluorescence intensity produced by miR-21 is 12.9-, 21.0-, and 12.3-fold of that

respectively produced by let-7a, let-7b, and let-7c (Fig. S6). These results suggest that this detection method for miRNAs has high sequence specificity to distinguish the perfectly complementary target and the mismatched sequences, and has promising application in single-nucleotide polymorphism analysis.

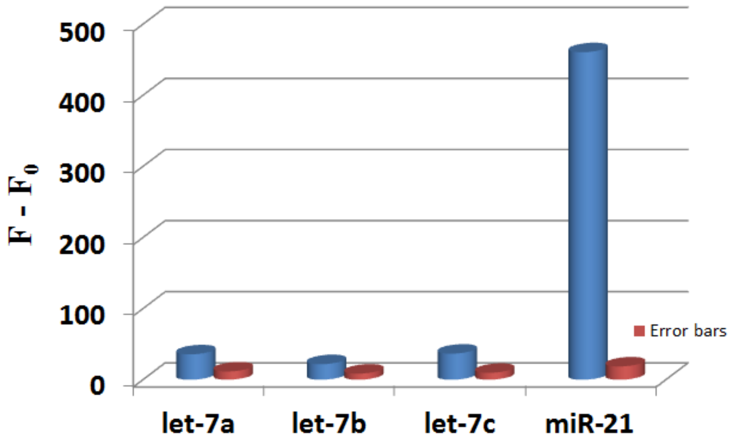

Fig. S6 Comparison of the fluorescence intensity produced by 5.0 × 10^{-10} M let-7a, let-7b, let-7c, and miR-21, where F and F_0 are fluorescence intensities of amplification products by THP-NAMAs method in the presence and absence of four different miRNAs, respectively. Error bars were estimated from three replicate measurements.

S6. Analysis of the cellular reaction product

The cellular reaction product was detected by the fluorescence intensity in the revised manuscript. Firstly, MCF-7 cells were incubated with culture medium containing DHS on GONPs (25 μg/mL) for 12 h, then were washed three times with PBS (0.01 M, pH 7.4). Secondly, the THP probe and circular-DNA fastened onto CMFs were incubated into the cells. The slides with fixed cells were incubated with 2% formamide for 30 min. After being washed three with PBS, the slides were dehydrated by a series of 70%, 80%, and 99% ice-cold ethanol for 3 min each and air-dried. Thirdly, the RCA reaction was performed in the humidified 37 ℃ incubator for 90 min with the reaction solution containing DEPC-treated water, 2 μL phi29 DNA polymerase and 6 μL dNTPs. Subsequently, 0.1 mol/L SDS solution was added to the mixture to attain an SDS concentration of 20 mmol/L.[S6] After these processed

cells on the slides were lyzed, the mixture was centrifuged (2000 r/min, 6 min) three times, and each supernatant was gathered. Fluorescence measurements of the reaction product were carried out on a F4600 fluorometer (Fig. S7). The regression equation of linear current response with MCF-7 cells is $F - F_0 = 136.6\ \lg N - 533.4$ with the correlation coefficient of 0.998 (F represent fluorescence intensities of MCF-7 cells; F_0 represent fluorescence intensities in the absence of MCF-7 cells; N represents the number of cells). The detection limit was determined to be 2×10^4 cells by three repetitive experiments.

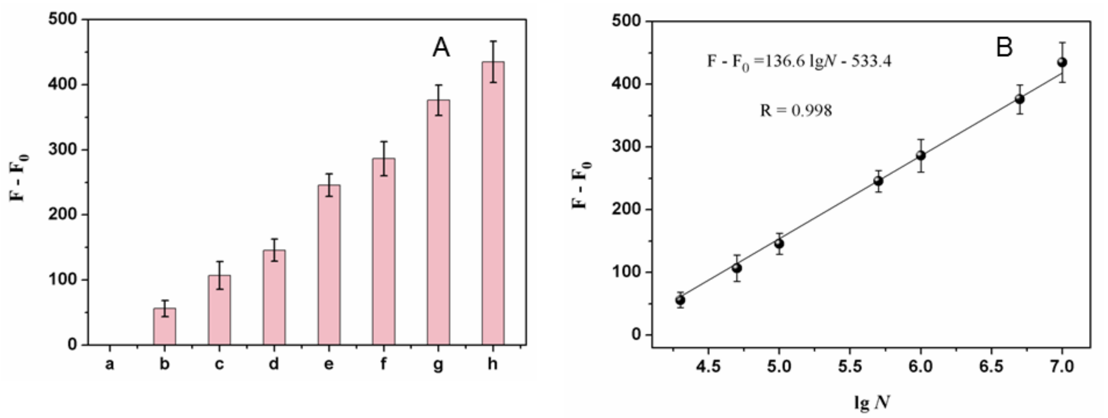

Fig. S7 (A) Fluorescence intensity changes of different amounts for MCF-7 cells, a-h: (a) 0, (b) 2×10^4, (c) 5×10^4, (d) 10^5, (e) 5×10^5, (f) 10^6, (g) 5×10^6, (h) 10^7 cells. (B) The corresponding calibration curve of fluorescence intensity *versus* the number of cells. The average of three spectra was acquired from different detection, and three repetitive experiments were carried out. Error bars displayed the standard deviation of three experiments. The blank was deducted from each value.

Table S1. miRNAs Sequences Used in This Work.

Note	Sequences(5'-3')
miR-21	5'-UAG CUU AUC AGA CUG AUG UUG A-3'
let-7a	5'-UGA GGU AGU AGG UUG UAU AGU U-3'
let-7b	5'-UGA GGU AGU AGG UUG UGU GGU U-3'
let-7c	5'-UGA GGU AGU AGG UUG UAU GGU U-3'

Table S2. DNA Sequences Used in This Work.

Note	Sequences
DNA1	5'-TCT CTC AGT TGT AGT CTT TTC TGA TGT TGA CTC TCT-3'
DNA2	5'-NH_2-AAA AGA GAG TCA ACA TCA GTC TGA TAA GCT A-3'
DNA3 (circular DNA)	5'-P- GAA CTC AGA GAG TCA ACA TCA GAA AAG ACT ACA ACT GAG AGA GAA GAA CTA CA TCC AGA CAT GAC GTA GT TT AG AGC-3'
DNA4	5'-CTC TGA GTT CGC TCT AA ACT AC-NH_2-3'
FAM-DNA5 (signal DNA)	5' - CTA CAT CCA GAC ATG ACG TAG - FAM- 3'
BHQ-DNA6 (3'-SH for preventing from side reaction of RCA）	5'- BHQ- CTA CGT CAT GTC TGG - SH-3'
FAM-DNA7	5'-FAM-AGA GAG TCA ACA TCA GTC TGA TAA GCT A-3'
BHQ-DNA8	5'-TCT CTC AGT TGT AGT CTT TTC TGA TGT TGA CTC TCT-BHQ-3'
BHQ-DNA9 (non-complementary)	5'-AAG GAA GAA CAC CCG TTT TTT GCC CAC AAG AAG GAA -BHQ-3'
FAM-DNA10	5'-TCT CTC AGT TGT AGT CTT TTC TGA TGT TGA CTC TCT -FAM-3'

References

[S1] K.C. Barick, S. Singh, N.V. Jadav, D. Bahadur, B.N. Pandey, P.A. Hassan, *Adv. Funct. Mater.* 2012, **22**, 4975-4985.

[S2] K.C. Barick, P.A. Hassan, J. Colloid Interface Sci. 2012, **369**, 96-102.

[S3] K. C. Barick , S. Singh, D. Bahadur, M. A. Lawande, D. P. Patkar, P. A. Hassan, *J. Colloid Interface. Sci.* 2014, **418**, 120-125.

[S4] A. Aravind, S. H. Varghese, S. Veeranarayanan, A. Mathew, Y. Nagaoka, S. Iwa, T. Fukuda,T. Hasumura, Y. Yoshida, T, Maekawa and D. Sakthi Kumar, Cancer Nano. 2012, **3**, 1-12.

[S5] A. Aravind, R. Nair, S. Raveendran, S. Veeranarayanan, Y. Nagaoka, T. Fukuda, T. Hasumura, H. Morimoto, Y. Yoshida, T. Maekawa and D. S. Kumar, J. Magn. Magn. Mater. 2013, **344**, 116-123.

[S6] L. Li, J. Feng, H. Liu, Q. Li, L. Tong and B. Tang, doi:10.1039/c5sc03909f.
